# Hypoxic cardiac fibroblasts from failing human hearts decrease cardiomyocyte beating frequency in an ALOX15 dependent manner

**DOI:** 10.1371/journal.pone.0202693

**Published:** 2018-08-23

**Authors:** Mikael Sandstedt, Victoria Rotter Sopasakis, Annika Lundqvist, Kristina Vukusic, Anders Oldfors, Göran Dellgren, Joakim Sandstedt, Lillemor Mattsson Hultén

**Affiliations:** 1 Department of Clinical Chemistry, Sahlgrenska University Hospital and Department of Clinical Chemistry and Transfusion Medicine, Institute of Biomedicine, Sahlgrenska Academy, University of Gothenburg, Gothenburg, Sweden; 2 Wallenberg Laboratory, Department of Molecular and Clinical Medicine, Institute of Medicine, Sahlgrenska Academy, University of Gothenburg, Gothenburg, Sweden; 3 Department of Pathology and Genetics, Institute of Biomedicine, Sahlgrenska Academy, University of Gothenburg, Gothenburg, Sweden; 4 Department of Cardiothoracic Surgery, Sahlgrenska University Hospital and Department of Molecular and Clinical Medicine, Institute of Medicine, Sahlgrenska Academy, University of Gothenburg, Gothenburg, Sweden; Niigata Daigaku, JAPAN

## Abstract

A common denominator for patients with heart failure is the correlation between elevated serum levels of proinflammatory cytokines and adverse clinical outcomes. Furthermore, lipoxygenase-induced inflammation is reportedly involved in the pathology of heart failure. Cardiac fibroblasts, which are abundant in cardiac tissue, are known to be activated by inflammation. We previously showed high expression of the lipoxygenase arachidonate 15 lipoxygenase (ALOX15), which catalyzes the conversion of arachidonic acid to 15-hydroxy eicosatetraenoic acid (15-HETE), in ischemic cardiac tissue. The exact roles of ALOX15 and 15-HETE in the pathogenesis of heart failure are however unknown. Biopsies were collected from all chambers of explanted failing human hearts from heart transplantation patients, as well as from the left ventricles from organ donors not suffering from chronic heart failure. Biopsies from the left ventricles underwent quantitative immunohistochemical analysis for ALOX15/B. Gene expression of ALOX enzymes, as well as 15-HETE levels, were examined in cardiac fibroblasts which had been cultured in either hypoxic or normoxic conditions after isolation from failing hearts. After the addition of fibroblast supernatants to human induced pluripotent stem cell-derived cardiomyocytes, intracellular calcium concentrations were measured to examine the effect of paracrine signaling on cardiomyocyte beating frequency. While ALOX15 and ALOX15B were expressed throughout failing hearts as well as in hearts from organ donors, ALOX15 was expressed at significantly higher levels in donor hearts. Hypoxia resulted in a significant increase in gene and protein expression of ALOX15 and ALOX15B in fibroblasts isolated from the different chambers of failing hearts. Finally, preconditioned medium from hypoxic fibroblasts decreased the beating frequency of human cardiomyocytes derived from induced pluripotent stem cells in an ALOX15-dependent manner. In summary, our results demonstrate that ALOX15/B signaling by hypoxic cardiac fibroblasts may play an important role in ischemic cardiomyopathy, by decreasing cardiomyocyte beating frequency.

## Introduction

Heart failure is a major cause of morbidity and mortality. While ischemic heart disease and hypertension are known to be the major causes of heart failure, underlying pathogenic mechanisms must be further elucidated to develop novel treatments. Human lipoxygenases are a family of lipid-peroxidizing enzymes that have been implicated in pathogenesis of ischemic heart disease [1, 2], heart failure [3, 4] and stroke [5, 6]. Arachidonate 15-lipoxygenase (ALOX15) catalyzes the conversion of arachidonic acid to 15-hydroxy eicosatetraenoic acid (15-HETE) [7]. Two enzymatic subtypes exist: type A (ALOX15) and type B (ALOX15B). We previously observed elevated levels of ALOX15 and 15-HETE in ischemic heart tissue from patients undergoing coronary artery bypass grafting (CABG) [8]. ALOX15B is increased in hypoxic human macrophages and symptomatic atherosclerotic carotid plaques [6, 9, 10].

The expression patterns of ALOX15 and ALOX15B in failing human hearts has however not been investigated previously. Hypoxic human cardiac endothelial cells, as well as cardiomyocytes derived from human induced pluripotent stem cells (hiPS-CMs), have previously been shown to express ALOX15 and 15-HETE [2]. Activated cardiac fibroblasts have been implicated in cardiac dysfunction as inflammatory response mediators after myocardial infarction, and fibroblast inflammatory signaling has been shown to contribute to cardiac disease [11, 12]. Due to the large amount of fibroblasts in the myocardium [13], lipoxygenase expression in cardiac fibroblasts could be an important pathophysiological mechanism in for example heart failure. Whether cardiac fibroblasts express ALOX15/B and contribute to 15-HETE signaling is however unknown. The effect of hypoxia, which is an essential factor in ischemic cardiomyopathy and resulting heart failure, is also unknown.

ALOX15/B and 15-HETE have several putative effects, including angiogenesis [14], chemotaxis [15, 16], inflammation, and extracellular matrix degradation [4, 17]. The effects of ALOX15/B on cardiomyocyte electrophysiology and heart rhythm are however unknown. This is of particular interest due to the role of cardiac fibroblasts in different forms of arrhythmia [18]. Indeed, fibrosis plays a central pathophysiological role in both atrial fibrillation [19] and ventricular tachycardia after myocardial infarction [20], and is also associated with sinus node dysfunction and bradycardia. While fibrosis may cause delayed electrical propagation and the formation of re-entry circuits, the role of paracrine signaling is less well understood. The effects of paracrine signaling of cardiac fibroblasts and ALOX15/B on heart rhythm should therefore be further explored.

Here, we assessed the expression of ALOX15/B in biopsies from failing human hearts as well as in donor hearts not suffering from chronic heart failure. We also investigated the effect of hypoxia on ALOX15/B gene and protein expression as well as 15-HETE generation in cardiac fibroblasts isolated from the four chambers of failing human hearts. The effect of paracrine signaling and ALOX15 on cardiomyocyte beating frequency was also investigated.

## Materials and methods

### Human material

Cardiac biopsies were obtained from explanted hearts of patients with severe heart failure undergoing heart transplantation at Sahlgrenska University Hospital. Biopsies were collected from all accessible heart chambers from six patients. Patient characteristics are shown in Table 1. As a control group for immunohistochemistry, additional biopsies were excised from the Left Ventricle (LV) of three organ donor hearts, not suitable for transplantation. Donors with known chronic heart failure were excluded. Clinical characteristics of included donors are summarized in Table 2. Ethical approval was obtained from the local Ethical Review Board at the University of Gothenburg. None of the transplant donors were from a vulnerable population and all donors or next of kin provided written informed consent that was freely given.

**Table 1:** Medical background of included heart transplantation patients.

Subject	Sex	Age	IHD	HF	LVAD	NYHA	LVEF (%)	Cause of HF	Other diseases
1	M	66	No	Yes	Yes	IIIA	20	Idiopathic DCM	Ventricular tachycardia, atrial fibrillation, previous stroke, diabetes type II, several episodes of salmonella sepsis
2	F	64	No	Yes	No	IIIB	20–30	Idiopathic DCM	Atrial fibrillation, diabetes type II, renal insufficiency, hypertension, previous malignancy, hypothyroidism
3	M	50	Yes	Yes	No	IIIA	35	Ischaemic DCM	Angina pectoris, previous CABG, diabetes type II, previous stroke, hypertension
4	F	60	No	Yes	No	IIIA	50	Cardiac Amyloidosis	Asthma, myeloma, PH
5	M	39	Yes	Yes	No	III	30	Ischaemic DCM	Previous MI with ventricular fibrillation, diabetes type I, diabetes retinopathy and nephropathy, PH
6	M	61	No	Yes	No	III	55	HCM due to prolonged atrial fibrillation	Ventricular tachycardia, atrial fibrillation, previous stroke, chronic obstructive pulmonary disease, renal insufficiency
7	M	67	Yes	Yes	Yes	IIIA	30	Ischaemic DCM	Previous MI, atrial fibrillation, tricuspid regurgitation, renal insufficiency, hypertension
8	M	65	No	Yes	No	IIIA	35	Idiopathic DCM	ASD, considered unrelated to HF. Atrial fibrillation. Asthma.

Table 1 summarizes the characteristics of the included patients at the time for heart transplantation. When treated with LVAD, the last recorded LVEF prior to LVAD implantation was noted. IHD = Ischaemic heart disease, HF = Heart failure, NYHA = New York Heart Association Functional Classification, LVEF = Left Ventricular Ejection Fraction, DCM = Dilated cardiomyopathy, HCM = Hypertrophic cardiomyopathy, CABG = Coronary artery bypass surgery, PH = Pulmonary hypertension, MI = Myocardial infarction, ASD = Atrial septum defect

**Table 2:** Medical background of included organ donors.

Subject	Sex	Age	History of HF	Cause of death	Reason for not meeting transplantation eligibility criteria	Other diseases
1	F	58	No	Intracerebral haemorrhage	IHD	Previous MI, atrial fibrillation, hypertension, psoriasis
2	F	44	No	Heart arrest due to hanging	Mitral valve regurgitation	
3	F	50	No	Intracerebral haemorrhage	IHD, takotsubo cardiomyopathy at time of organ extraction	Previous MI, ventricular tachycardia

Table 2 summarizes the characteristics of the included organ donors at the time of death. IHD = Ischaemic heart disease, HF = Heart failure, MI = Myocardial infarction

### Immunohistochemistry

Biopsies were attached onto a cork disc, embedded in the tissue-mounting reagent Tragacant (Histolab, Gothenburg, Sweden), snap frozen in liquid nitrogen, and preserved at -80°C until cryosectioning. Frozen tissue was sectioned into 7-μm serial sections using a cryotome. Immunohistochemistry (IHC) was conducted using 7-μm cryosections of left ventricular tissue. Frozen tissue sections were fixed in -20°C acetone for 10 min, washed with phosphate-buffered saline (PBS) solution, and blocked in PBS containing 2% bovine serum albumin (BSA, Sigma-Aldrich, St. Louis, MO), 0.3% Triton–X100 (Sigma-Aldrich), and 5% goat serum (Thermo Fisher Scientific, Waltham, MA, USA) for 30 minutes at room temperature. Samples were stained using ALOX15 mouse IgG2b monoclonal (ab119774, Abcam, Cambridge, UK) or ALOX15B rabbit IgG polyclonal (ab23691, Abcam) antibodies diluted in Tris-buffered saline with 1% BSA. Corresponding isotype controls for the primary antibodies were used for determining background staining. Results were visualized by staining with secondary antibodies: goat anti-mouse IgG Alexa Fluor 647 (A11032, Thermo Fisher Scientific) and donkey anti-rabbit IgG Alexa Fluor 546 (A10040, Thermo Fisher Scientific) for ALOX15B, diluted in PBS with 1% BSA. Samples were mounted with prolong gold antifade with DAPI (Thermo Fisher Scientific).

### Image analysis and quantification of the ALOX15 and ALOX15B staining

Large images were acquired by using a Nikon ECLIPSE Ti fluorescence microscope (Nikon Corporation, Tokyo, Japan) with an ANDOR Zyla camera (Andor Technology, Belfast, UK). The images were scanned automatically using a motorized board and the "stitch images" option provided in the software. The size of the images was 7X7 fields of view using a 20x objective. Images were acquired at 3 Z-levels to capture all the nuclei in focus. Images were exported to Image J software (v. 1.52d, Fiji distribution) for further analysis [21]. To reduce background noise, an intensity threshold was set for each staining using a customized plugin (S1 File). Pixel values below the threshold were set to zero. The threshold was then subtracted from pixel values above the threshold in order to get a continuous distribution of pixel values. All images, including isotype controls, were treated equally.

Region of interests were created to include most of the stained tissue, but excluding obvious staining artefacts. Mean intensity measurements were carried out on ALOX15 and ALOX15B expression, respectively. To correct for background staining, these values were subtracted with corresponding mean intensity values for isotype controls.

### Primary isolation of non-myocyte cells

Biopsies were collected in cold PBS, rinsed with PBS to remove residual blood, weighed, and cut into small pieces. Tissue pieces were digested with 0.52 Wünsch units/mL Liberase type TM (Roche, Basel, Switzerland) and 0.05 mg/ml DNase-I (Roche) in DMEM/F12 (Thermo Fisher Scientific) at 37°C for 4.5 h with magnetic stirring. After washing once, samples were further incubated for 10 min in 0.05% Tryspin-EDTA (Thermo Fisher Scientific). Cells were then resuspended in DMEM/F12 supplemented with 10% fetal bovine serum (FBS, Sigma-Aldrich), and filtered sequentially through 250-μm and 100-μm cell strainer filters (BD, Franklin Lakes, NJ) to remove residual tissue fragments and cardiomyocytes. The remaining cell suspension was centrifuged and then seeded as a monolayer culture on Primaria culture plates (BD) at a density of approximately 1–2 mg tissue/cm^2^.

### Fibroblast cell culture

Isolated fibroblasts were cultured in growth medium consisting of DMEM/F12 supplemented with 10% pooled human serum, penicillin/streptomycin (PEST, Thermo Fisher Scientific) and L-glutamine (Thermo Fisher Scientific). To mimic the effect of ischemia, fibroblasts were cultured in hypoxic (1% oxygen) or normoxic (21% oxygen) conditions for 24 h before analysis with or without the specific human ALOX15 type A inhibitor ML351 (10 μmol/L, kindly provided by Professor Theodore Holman of the Department of Chemistry and Biochemistry, University of California−Santa Cruz, USA) [22, 23] or the less specific human ALOX12 and ALOX15 inhibitor baicalein (10 μmol/L Sigma-Aldrich) [24, 25].

### RT-qPCR

Total RNA was isolated from cultured fibroblasts (n = 3–6 for the different heart chambers) using an RNeasy Kit (Qiagen, Hilden, Germany). cDNA synthesis was performed using a cDNA Reverse Transcription Kit (Applied Biosystems, Foster City, CA) with standard protocols for the Gene Amp 9700 PCR System (Applied Biosystems). The following TaqMan gene expression assays from Qiagen were used: *ALOX15* (Hs00609608_m1), *ALOX15B* (Hs00153988_m1), and *HPRT1* (Hs99999909_m1). The standard protocol for the ABI Prism 7900 HT sequence detection system (Applied Biosystems) was run for 40 amplification cycles. The relative comparative method was used to analyze reverse transcriptase quantitative PCR (RT-qPCR) data [26] with *HPRT1* as a reference gene [27].

### Immunocytochemistry

Immunocytochemistry was conducted using formalin-fixed fibroblasts exposed to normoxic or hypoxic conditions. Samples were stained and visualized in the same manner as described for IHC.

### Quantification of HETE concentrations

15-HETE concentrations were analyzed in cell lysates of harvested cultured fibroblasts by enzyme-linked immunosorbent assay with the 15-HETE ELISA Kit (ab133035, Abcam) according to the manufacturer’s protocol. n = 3–6 for all chambers and treatments except for ML351 treated hypoxic fibroblasts from right atrium (n = 2).

### Measurements of hiPS-CMs beating frequency

Cardiomyocytes derived from human induced pluripotent stem cells (hiPS-CMs, Takara Bio USA, Madison, WI) were cultured according to the manufacturer’s protocol for 5 days. Cultures were spontaneously contracting prior to experiments. Intracellular Ca^2+^ concentrations of beating hiPS-CMs was analyzed using the Fura-2 QBT fluorescence-based calcium indicator and EarlyTox Cardiotoxicity Kit (Molecular Devices, Sunnyvale, CA) to accurately measure calcium mobilization. As cardiomyocytes contract, signal traces show an increase in fluorescent signal corresponding to the increased cytoplasmic Ca^2+^ concentration when Ca^2+^ is released from the sarcoplasmic reticulum. The signal decrease as Ca^2+^ is transported back into the sarcoplasmic reticulum as part of the cell relaxation process. The frequency of signal traces corresponds to cardiomyocyte beating frequency.

Pre-conditioned medium was collected from fibroblasts isolated from the right atrium (n = 3), exposed to normoxia or hypoxia, with or without the addition of ML351 or baicalein. hiPS-CMs were incubated for two hours in a mixture of 100 μL hiPS-CM culture medium and 100 μL calcium probe. Next, 100 μL of medium-dye mixture was replaced with 100 μL of pre-conditioned fibroblast medium. Intracellular calcium concentrations were measured after 0, 30, 60, and 120 minutes after the addition of pre-conditioned medium. Each well was registered for 50 sec to determine the beating frequency of hiPS-CMs. Measurements were conducted using a SpectraMax i3 microplate reader (Molecular Devices) according to the manufacturer’s instructions. Changes in Ca^2+^ fluorescent signal were registered as relative fluorescence units (RFU), corresponding to the hiPS-CM beating frequency.

### Statistics

In order to achieve a near normal data distribution without heteroscedasticity, log transformation was performed for all data, except for mean fluorescence intensities, prior to statistical analysis. Group wise comparisons of mean fluorescence intensities were carried out using two-sided student's T-tests. Pairwise comparisons of gene expression levels between fibroblasts incubated under normoxia and hypoxia were carried out using two-sided paired samples T-tests for each heart chamber of origin. Two-way ANOVA analyses were performed for 15-HETE concentrations. One-way ANOVA analysis was performed for each time point for beating frequencies. For all ANOVA analyses, experiment number was assigned as a random block factor and Tukey’s test was used to correct for mass significance. For all statistical analyses, a P value < 0.05 was considered significant. Analyses were carried out using SPSS v. 20 (IBM, New York, NY, USA), MS Excel (Microsoft, Redmond, WA, USA) or Graph Pad Prism v. 7.04 (GraphPad Software, La Jolla, CA, USA). Data are presented as mean and standard error of the mean (SEM), unless otherwise stated. For 15-HETE measurements, data were normalized to the corresponding normoxic control for presentation. Graphs and box plots were drawn up using Graph Pad Prism v. 7.04.

## Results

### ALOX15 and ALOX15B are expressed in the failing human heart

**Fig 1:**
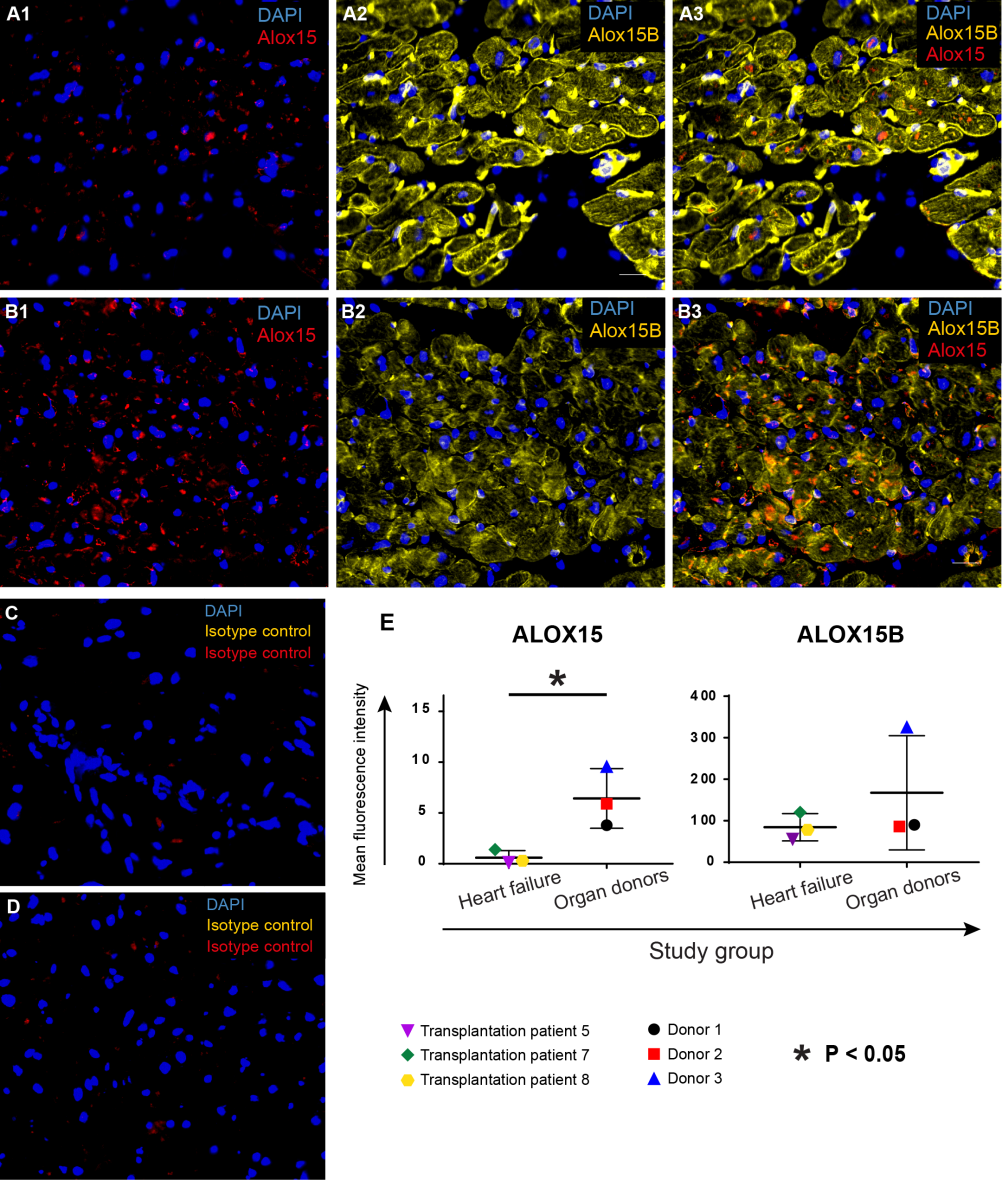
Expression of ALOX15 and ALOX15B in left ventricular tissue from failing human hearts and donor hearts. Biopsies were snap frozen with liquid nitrogen and processed for IHC staining. Slides were incubated with primary antibodies for ALOX15 (red) and ALOX15B (yellow), followed by incubation with fluorochrome-conjugated secondary antibodes and mounting with DAPI (blue). (A) Representative images of ALOX15 and ALOX15B expression in the failing human heart. (B) Representative images of ALOX15 and ALOX15B in donor hearts not suffering from chronic heart failure. Corresponding isotype controls are shown for a failing heart (C) as well as for a donor heart (D). (E) Quantification of ALOX15 and ALOX15B expression. Both heart failure patients and donors expressed ALOX15 and ALOX15B, with a significantly higher expression of ALOX15 in donor hearts. Group wise statistical comparisons were performed using two-sided Student’s T-tests.

Left ventricular tissue from patients undergoing heart transplantation surgery was subjected to IHC analysis for ALOX15/B. As a control group, donor hearts without chronic heart failure was used. ALOX15 and ALOX15B were expressed throughout the myocardium in both failing and donor hearts (Fig 1A–1D). ALOX15B seemed to be more abundantly expressed and partly co-localized with ALOX15 expression. Quantification of the staining confirmed expression of both ALOX15 and ALOX15B in both failing hearts and donor hearts. For ALOX15, the expression was significantly higher in the donor group.

### Hypoxia induces ALOX15/ALOX15B expression in cardiac fibroblasts

**Fig 2:**
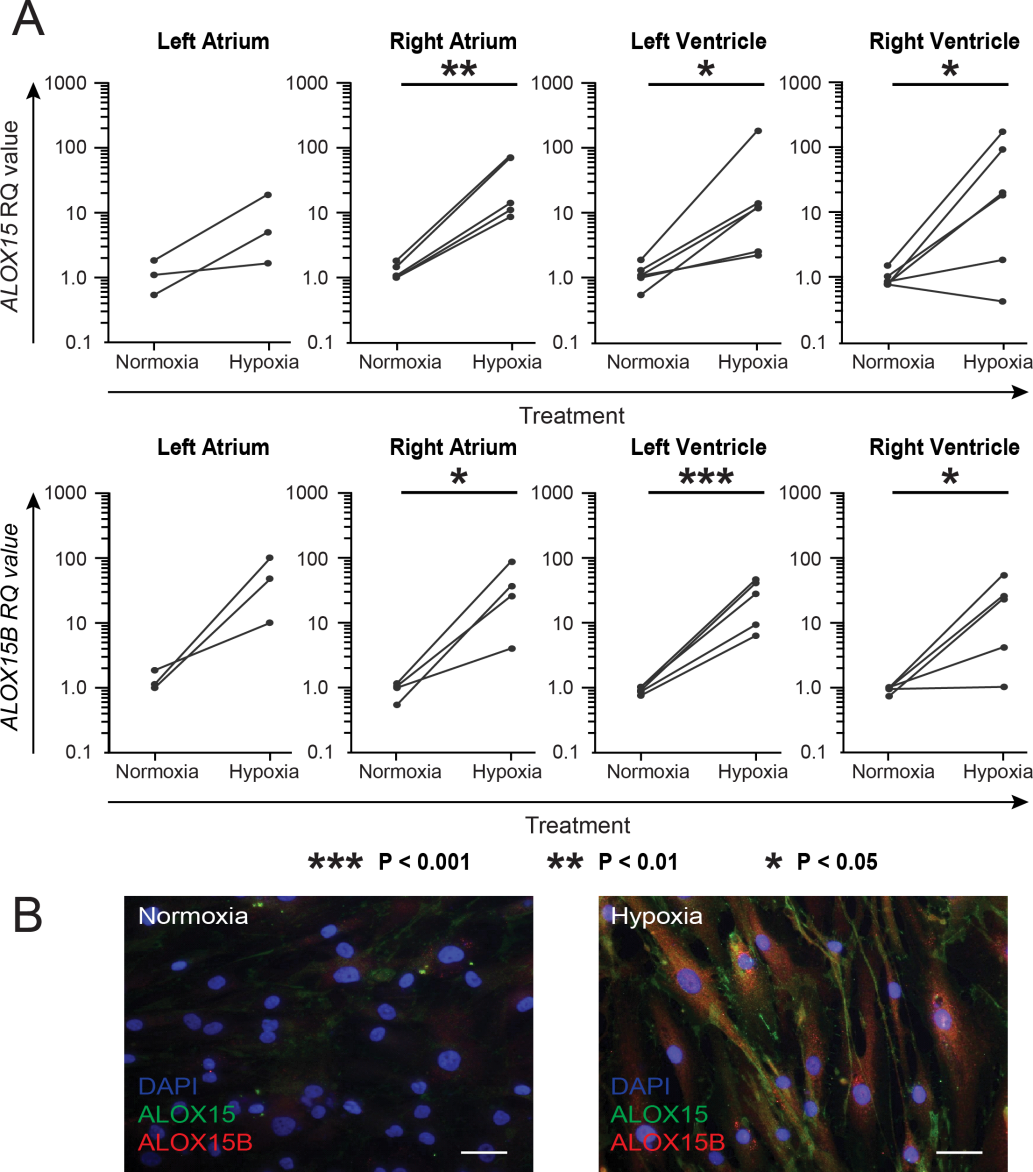
Expression of ALOX15 and ALOX15B in hypoxic human cardiac fibroblasts. Cardiac fibroblasts were isolated from left (n = 3) and right (n = 5) atrium as well as left (n = 6) and right (n = 6) ventricle and subjected to 1% or 21% oxygen for 24 h. (A) Harvested cells were subjected to RT-qPCR and paired T-tests was performed using log-transformed RQ values. Paired RQ values are demonstrated as dots combined by a line. *** p < 0.001, **p < 0.01, *p < 0.05. (B) Immunocytochemical staining of human cardiac fibroblasts with primary antibodies against ALOX15 (green), ALOX15B (red) followed by flurochrome-conjugated secondary antibodies and DAPI (blue). Representative images of normoxic and hypoxic fibroblasts are shown. Scale bar = 40 μM.

To study whether fibroblasts express ALOX15/B as well as the effect of hypoxia, fibroblasts were isolated from the four chambers of failing human hearts. Fibroblasts were cultured in vitro in hypoxia or normoxia for 24 h, followed by harvesting and gene expression analysis by RT-qPCR. Hypoxia resulted in significant increases in *ALOX15* and *ALOX15B* gene expression for fibroblasts from all chambers except for the left atrium (Fig 2A). The expression of *ALOX15* as well as *ALOX15B* also tended to increase for fibroblasts isolated from the left atrium. Immunocytochemical staining of hypoxic fibroblasts demonstrated an increased protein expression of both ALOX15 and ALOX15B as compared to normoxic fibroblasts (Fig 2B).

### The specific ALOX15 inhibitor ML351 decreased 15-HETE levels in cardiac fibroblasts

**Fig 3:**
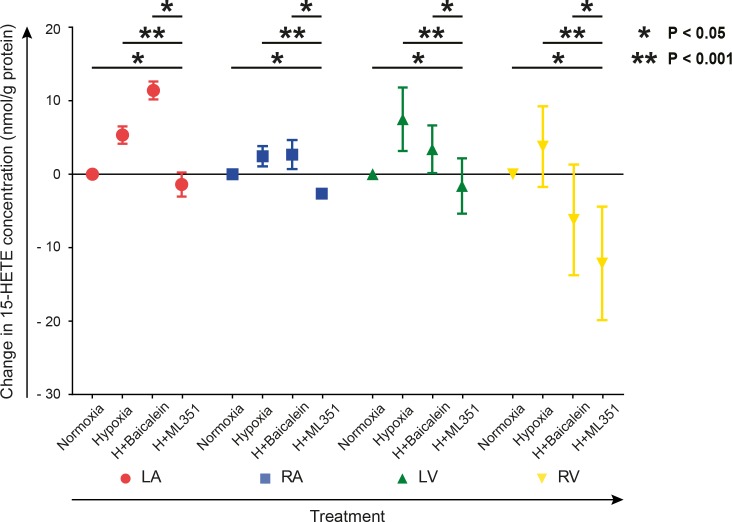
15-HETE levels in cardiac fibroblasts under different oxygen tensions and ALOX15 inhibition. Cardiac fibroblasts isolated from left (LA) and right (RA) atrium as well as left (LV) and right (RV) ventricle were subjected to 1% or 21% oxygen for 24 h. Hypoxic fibroblasts were cultured with or without the specific ALOX15 inhibitor ML351 (10 μmol/L) or the less specific ALOX12 and ALOX15 inhibitor baicalein (10 μmol/L). n = 5 for every chamber in the hypoxic and normoxic groups. n = 3 for every chamber in the inhibitor groups, except for ML351 treated fibroblasts from right atrium (n = 2). Two-way ANOVA was performed with Tukey's multiple comparisons test using log-transformed data. Concentrations were then normalized by subtracting the normoxic controls and means were calculated for demonstration. * p < 0.05, ** p < 0.001.

Cell lysates of normoxic and hypoxic fibroblasts, cultured with or without addition of the specific ALOX15 inhibitor ML351 or the less specific ALOX12 and ALOX15 inhibitor baicalein, were collected. Levels of 15-HETE were then analyzed. Concentrations of 15-HETE tended to increase under hypoxia (Fig 3). Addition of ML351 to hypoxic fibroblasts resulted in a significant reduction in 15-HETE concentrations as compared to normoxic and hypoxic fibroblasts respectively. This effect was not observed for baicalein.

### Paracrine signaling by hypoxic cardiac fibroblasts decreases the beating frequency of hiPS-CMs in an ALOX15-dependent manner

**Fig 4:**
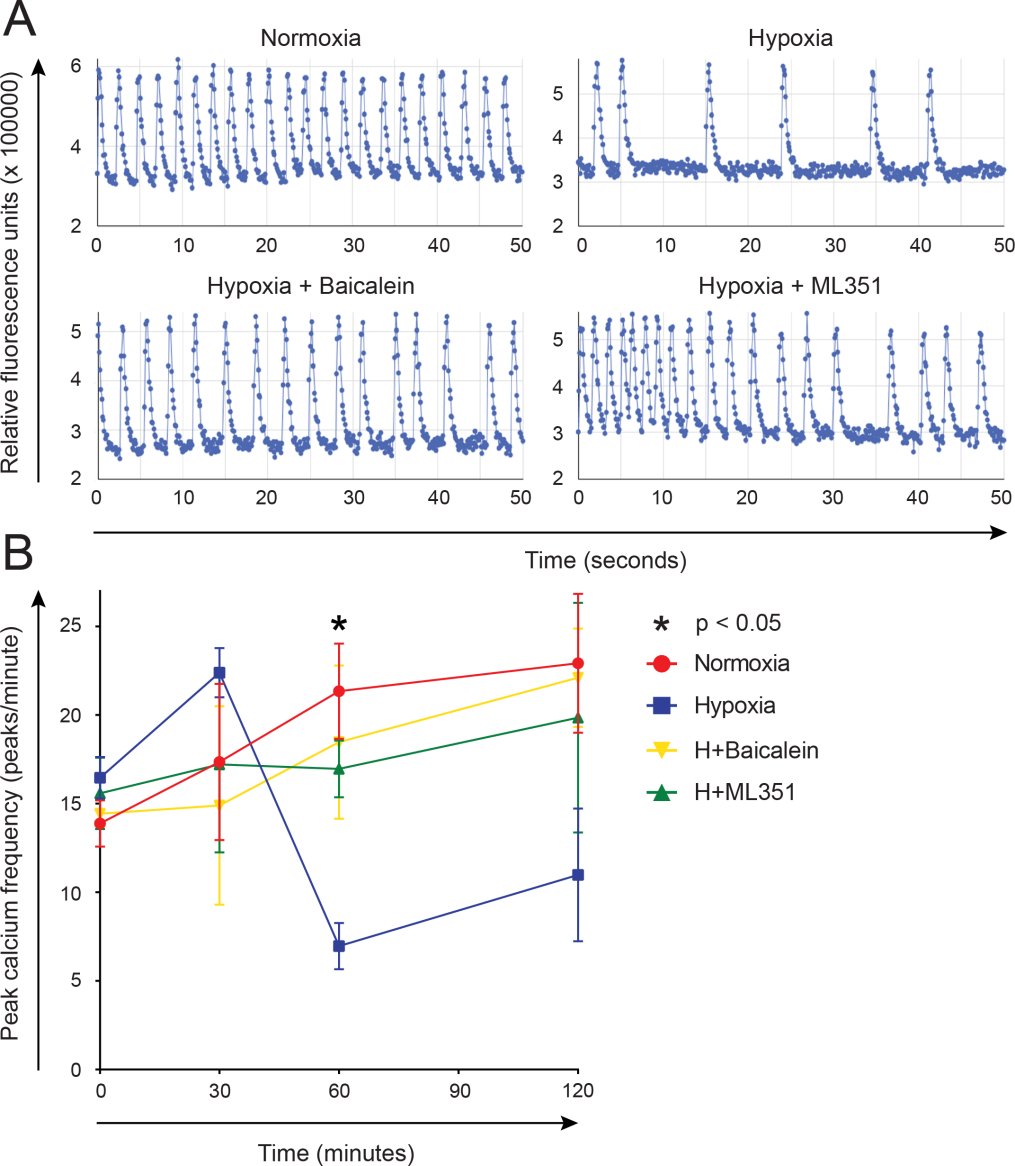
Preconditioned medium from hypoxic human cardiac fibroblasts affected cardiomyocyte beating frequency. Cardiomyocytes derived from human induced pluripotent stem cells were cultured with preconditioned medium from human cardiac fibroblasts subjected to 1% or 21% oxygen with or without the addition of ML351 or baicalein, and calcium probe solution. Intracellular calcium concentrations were registered as relative fluorescence units (RFU) for up to 120 minutes and beating frequency was determined. (A) Representative images of changes in RFU after 60 minutes incubation with preconditioned medium. (B) Changes in beating frequency for different time points. One-way ANOVA was performed for each time point with Tukey's multiple comparisons test using log-transformed data. Data is demonstrated as mean ± SEM. * p < 0.05 for hypoxia vs. normoxia, baicalein and ML351 at 60 minutes.

To examine the paracrine effects of fibroblasts on hiPS-CM beating frequency under different oxygen tensions, isolated human cardiac fibroblasts were cultured under 1% or 21% oxygen for 24 h. ML351 or baicalein was added to some hypoxic cultures. Preconditioned medium from the fibroblasts was then mixed with calcium probe solution and added to beating hiPS-CMs. Calcium concentrations were quantified as relative fluorescence units (RFU) at different time points (Fig 4A), and beating frequency determined. The beating frequency was significantly decreased after 1 h when preconditioned medium from hypoxic fibroblasts had been added, as compared to medium from normoxic fibroblasts (Fig 4B). Addition of ML351 or baicalein resulted in a significantly higher frequency as compared to medium from hypoxic fibroblasts without inhibitors. These data suggest that hypoxic fibroblasts may decrease hiPS-CM beating frequency through paracrine signaling in an ALOX15 dependent manner.

## Discussion

Human lipoxygenases are lipid peroxidizing enzymes which have been shown to contribute to heart failure [3, 4]. For example, Kayama et al. observed macrophage infiltration and lipoxygenase-induced inflammation in the development of heart failure [3]—demonstrating a link between lipoxygenases and inflammation. Most of the knowledge is however based on studies in animal models. While we previously have observed a high expression of ALOX15 and 15-HETE in ischemic heart tissue [2, 8], the expression patterns of ALOX15 and ALOX15B in failing human hearts have not been known. In the present study, we demonstrate for the first time that ALOX15 and ALOX15B is expressed in failing human hearts, and that ALOX15/B signaling therefore may contribute to human heart failure. While ALOX15B was expressed at similar levels in failing and donor hearts, ALOX15 was expressed at lower levels. This was an unexpected finding, as lipoxygenases have been reported to increase in mice suffering from diabetic cardiomyopathy as well as in rats suffering from hypertensive heart failure [3, 4]. As lipoxygenase expression patterns may be species dependent [28], these varying results could be explained by differences between rodent and human hearts. Another possible explanation is that ALOX15 expression patterns depend on the etiology of heart failure. In the present study, two of the failing hearts suffered from ischemic cardiomyopathy, while the third suffered from idiopathic dilated cardiomyopathy. Notably, two of the donors also suffered from myocardial ischemia, indicating that the decrease in ALOX15 expression indeed was associated with heart failure and not myocardial ischemia in itself.

Fibroblasts constitute a heterogeneous cell population that constitutes an important fraction of the non-cardiomyocytes of the heart [13]. Due to their proximity to cardiomyocytes as well as vasculature, fibroblasts may act as a key player in heart disease through paracrine signaling. Fibroblasts have for example been implicated in the progress of heart failure [29]. Besides causing myocardial fibrosis they may also act as proinflammatory supporter cells [11]. Whether cardiac fibroblasts contribute to heart failure or other cardiac pathologies through ALOX15/B signaling has however not been described. Our results demonstrate on gene as well as protein level that fibroblasts isolated from either of the four chambers of failing human hearts express ALOX15 as well as ALOX15B in vitro. As hypoxia resulted in an increased expression of *ALOX15* and *ALOX15B*, fibroblasts might constitute an important source for the previously observed increase of *ALOX15/B* expression in the ischemic myocardium [2].

The concentrations of 15-HETE have previously been shown to be elevated in myocardial tissue from patients undergoing CABG as compared to patients undergoing aortic valve replacement surgery, suggesting an increased 15-HETE signaling in ischemic myocardium [2]. Due to the elevated gene expression of *ALOX15* and *ALOX15B* in fibroblasts under hypoxia, a similar elevation in 15-HETE levels in conditioned media would therefore be expected. Hypoxia indeed tended to result in elevated 15-HETE levels. As the specific ALOX15 inhibitor ML351 negated this effect, ALOX15 type A seems to be an important source for 15-HETE. within fibroblasts.

Considering the well-described role of association of ischemic cardiomyopathy, fibrosis and arrhythmia [18–20], the increase in ALOX15/B under hypoxia suggested that fibroblasts might affect cardiomyocyte electrophysiology via ALOX15/B signaling. Human cardiomyocytes were therefore incubated with preconditioned media of normoxic and hypoxic fibroblasts respectively. Our results show that paracrine signaling by hypoxic cardiac fibroblasts result in a decreased cardiomyocyte beating frequency. Interestingly, the addition of ALOX15 inhibitors ML351 and baicalein negated the effect of hypoxia, demonstrating that the effect was at least in part dependent on ALOX15 signaling. Surprisingly, these paracrine effects suggest that hypoxic fibroblasts may act in an anti-arrhythmic manner by lowering cardiomyocyte chronotropy. In an in vivo rat model of coronary artery occlusion, baicalein was found to inhibit arrhythmias induced by ischemia-reperfusion injury [30]. The effects of hypoxic fibroblasts are further relevant as even a moderate increase in heart rate in patients suffering from ischemic heart disease has been shown to result in negative prognosis [31]. The importance of heart rate in heart failure with reduced ejection fraction has also been shown in the SHIFT-trial, in which treatment with the selective sinoatrial node inhibitor Ivabradine resulted in decreased cardiovascular mortality and hospital admissions due to heart failure [32]. Fibroblasts might therefore have a positive paracrine effect on heart function, particularly in the setting of ischemic cardiomyopathy.

Some limitations of the present study should be acknowledged. Cardiac tissue for isolation of cardiac fibroblasts was only available from patients with severe heart failure undergoing heart transplantation. It is therefore not known to what extent cellular function is affected by cardiac disease. Furthermore, the number of patients included in the study is small, due to the limited number of suitable individuals for enrollment in the study. As the left and/or right atrium often at least in part is used and not removed during transplantation surgery, fibroblasts could not be isolated from the atria of some patients. While ML351 and baicalein both have been described to inhibit ALOX15, there exists no specific inhibitor of ALOX15B. The contribution of ALOX15B to 15-HETE signaling and the observed effects on cardiomyocyte beating frequency could therefore not be assessed. Finally, hiPS-CMs were used as a model system in order to mimic the phenotype of adult human cardiomyocytes and pacemaker cells. hiPS-CMs share many important ion currents and traits with adult cardiomyocytes [33]. Possible differences between relatively immature hiPS-CMs and mature cardiomyocytes makes extrapolation of findings to mature cardiomyocytes as well as pacemaker cells more difficult.

In summary, our results demonstrate the expression of ALOX15 and ALOX15B in failing as well as in donor hearts. ALOX15/B signaling may play an important role in heart disease, including heart failure. Isolated fibroblasts from all four chambers of the heart expressed ALOX15 as well as ALOX15B, and gene expression levels were further increased under hypoxia. Fibroblasts may be of particular importance in ischemic cardiomyopathy, for example by decreasing cardiomyocyte beating frequency through paracrine signaling. Due to the possible therapeutic applications, the role of fibroblasts and ALOX15/B signaling in heart failure should be subject for further studies.

## Supporting information

S1 File Source code of thresholding plugin used in IHC analysis. (XLSX)

## References

[1] Song L, Yang H, Wang HX, Tian C, Liu Y, Zeng XJ, et al Inhibition of 12/15 lipoxygenase by baicalein reduces myocardial ischemia/reperfusion injury via modulation of multiple signaling pathways. Apoptosis: an international journal on programmed cell death. 2014;19(4):567–80. Epub 2013/11/20. 10.1007/s10495-013-0946-z ; PubMed Central PMCID: PMC25_Inflammation. 24248985

[2] Lundqvist A, Sandstedt M, Sandstedt J, Wickelgren R, Hansson GI, Jeppsson A, et al The Arachidonate 15-Lipoxygenase Enzyme Product 15-HETE Is Present in Heart Tissue from Patients with Ischemic Heart Disease and Enhances Clot Formation. PLoS ONE. 2016;11(8):e0161629 10.1371/journal.pone.0161629 ; PubMed Central PMCID: PMCPMC4994938. 27552229 PMC4994938

[3] Kayama Y, Minamino T, Toko H, Sakamoto M, Shimizu I, Takahashi H, et al Cardiac 12/15 lipoxygenase-induced inflammation is involved in heart failure. J Exp Med. 2009;206(7):1565–74. 10.1084/jem.20082596 ; PubMed Central PMCID: PMCPMC2715088. 19546247 PMC2715088

[4] Suzuki H, Kayama Y, Sakamoto M, Iuchi H, Shimizu I, Yoshino T, et al Arachidonate 12/15-lipoxygenase-induced inflammation and oxidative stress are involved in the development of diabetic cardiomyopathy. Diabetes. 2015;64(2):618–30. 10.2337/db13-1896 . 25187369

[5] Lapchak PA, Maher P, Schubert D, Zivin JA. Baicalein, an antioxidant 12/15-lipoxygenase inhibitor improves clinical rating scores following multiple infarct embolic strokes. Neuroscience. 2007;150(3):585–91. 10.1016/j.neuroscience.2007.09.033 . 17942241

[6] Gertow K, Nobili E, Folkersen L, Newman JW, Pedersen TL, Ekstrand J, et al 12- and 15-lipoxygenases in human carotid atherosclerotic lesions: associations with cerebrovascular symptoms. Atherosclerosis. 2011;215(2):411–6. Epub 2011/02/15. 10.1016/j.atherosclerosis.2011.01.015 ; PubMed Central PMCID: PMC25_Inflammation. 21316676

[7] Haeggstrom JZ, Funk CD. Lipoxygenase and Leukotriene Pathways: Biochemistry, Biology, and Roles in Disease. Chem Rev. 2011;111(10):5866–98. 10.1021/cr200246d PubMed PMID: WOS:000296001000003. 21936577

[8] Magnusson LU, Lundqvist A, Asp J, Synnergren J, Johansson CT, Palmqvist L, et al High expression of arachidonate 15-lipoxygenase and proinflammatory markers in human ischemic heart tissue. Biochem Biophys Res Commun. 2012;424(2):327–30. 10.1016/j.bbrc.2012.06.117 . 22750246

[9] Magnusson LU, Lundqvist A, Karlsson MN, Skalen K, Levin M, Wiklund O, et al Arachidonate 15-lipoxygenase type B knockdown leads to reduced lipid accumulation and inflammation in atherosclerosis. PLoS ONE. 2012;7(8):e43142 Epub 2012/08/23. 10.1371/journal.pone.0043142 ; PubMed Central PMCID: PMC3422220. 22912809 PMC3422220

[10] Hulten LM, Olson FJ, Aberg H, Carlsson J, Karlstrom L, Boren J, et al 15-Lipoxygenase-2 is expressed in macrophages in human carotid plaques and regulated by hypoxia-inducible factor-1alpha. Eur J Clin Invest. 2010;40(1):11–7. 10.1111/j.1365-2362.2009.02223.x . 19912316

[11] Lindner D, Zietsch C, Tank J, Sossalla S, Fluschnik N, Hinrichs S, et al Cardiac fibroblasts support cardiac inflammation in heart failure. Basic Res Cardiol. 2014;109(5):428 10.1007/s00395-014-0428-7 . 25086637

[12] Frangogiannis NG. The inflammatory response in myocardial injury, repair, and remodelling. Nature reviews Cardiology. 2014;11(5):255–65. 10.1038/nrcardio.2014.28 ; PubMed Central PMCID: PMCPMC4407144. 24663091 PMC4407144

[13] Pinto AR, Ilinykh A, Ivey MJ, Kuwabara JT, D'Antoni ML, Debuque R, et al Revisiting Cardiac Cellular Composition. Circ Res. 2016;118(3):400–9. 10.1161/CIRCRESAHA.115.307778 ; PubMed Central PMCID: PMCPMC4744092. 26635390 PMC4744092

[14] Kundumani-Sridharan V, Niu J, Wang D, Van Quyen D, Zhang Q, Singh NK, et al 15(S)-hydroxyeicosatetraenoic acid-induced angiogenesis requires Src-mediated Egr-1-dependent rapid induction of FGF-2 expression. Blood. 2010;115(10):2105–16. 10.1182/blood-2009-09-241802 ; PubMed Central PMCID: PMCPMC2837334. 20053757 PMC2837334

[15] Reilly KB, Srinivasan S, Hatley ME, Patricia MK, Lannigan J, Bolick DT, et al 12/15-Lipoxygenase activity mediates inflammatory monocyte/endothelial interactions and atherosclerosis in vivo. J Biol Chem. 2004;279(10):9440–50. 10.1074/jbc.M303857200 . 14676201

[16] Danielsson KN, Rydberg EK, Ingelsten M, Akyurek LM, Jirholt P, Ullstrom C, et al 15-Lipoxygenase-2 expression in human macrophages induces chemokine secretion and T cell migration. Atherosclerosis. 2008;199(1):34–40. 10.1016/j.atherosclerosis.2007.10.027 . 18067895

[17] Wu MY, Lin TH, Chiu YC, Liou HC, Yang RS, Fu WM. Involvement of 15-lipoxygenase in the inflammatory arthritis. J Cell Biochem. 2012;113(7):2279–89. Epub 2012/05/11. 10.1002/jcb.24098 . 22573549

[18] Miragoli M, Salvarani N, Rohr S. Myofibroblasts induce ectopic activity in cardiac tissue. Circ Res. 2007;101(8):755–8. 10.1161/CIRCRESAHA.107.160549 . 17872460

[19] Spach MS. Mounting evidence that fibrosis generates a major mechanism for atrial fibrillation. Circ Res. 2007;101(8):743–5. 10.1161/CIRCRESAHA.107.163956 . 17932329

[20] Ripplinger CM, Lou Q, Li W, Hadley J, Efimov IR. Panoramic imaging reveals basic mechanisms of induction and termination of ventricular tachycardia in rabbit heart with chronic infarction: implications for low-voltage cardioversion. Heart Rhythm. 2009;6(1):87–97. 10.1016/j.hrthm.2008.09.019 ; PubMed Central PMCID: PMCPMC2650268. 18996057 PMC2650268

[21] Schindelin J, Arganda-Carreras I, Frise E, Kaynig V, Longair M, Pietzsch T, et al Fiji: an open-source platform for biological-image analysis. Nat Methods. 2012;9(7):676–82. 10.1038/nmeth.2019 ; PubMed Central PMCID: PMCPMC3855844. 22743772 PMC3855844

[22] van Leyen K, Holman TR, Maloney DJ. The potential of 12/15-lipoxygenase inhibitors in stroke therapy. Future Med Chem. 2014;6(17):1853–5. 10.4155/fmc.14.129 ; PubMed Central PMCID: PMC4280907. 25495979 PMC4280907

[23] Rai G, Joshi N, Jung JE, Liu Y, Schultz L, Yasgar A, et al Potent and selective inhibitors of human reticulocyte 12/15-lipoxygenase as anti-stroke therapies. J Med Chem. 2014;57(10):4035–48. 10.1021/jm401915r ; PubMed Central PMCID: PMC4033661. 24684213 PMC4033661

[24] van Leyen K, Kim HY, Lee SR, Jin G, Arai K, Lo EH. Baicalein and 12/15-lipoxygenase in the ischemic brain. Stroke. 2006;37(12):3014–8. 10.1161/01.STR.0000249004.25444.a5 . 17053180

[25] Deschamps JD, Kenyon VA, Holman TR. Baicalein is a potent in vitro inhibitor against both reticulocyte 15-human and platelet 12-human lipoxygenases. Bioorganic & medicinal chemistry. 2006;14(12):4295–301. Epub 2006/02/28. 10.1016/j.bmc.2006.01.057 ; PubMed Central PMCID: PMC3_ Growth Factors, markers and signal pathways. 16500106

[26] Livak KJ, Schmittgen TD. Analysis of relative gene expression data using real-time quantitative PCR and the 2(-Delta Delta C(T)) Method. Methods (San Diego, Calif. 2001;25(4):402–8. 10.1006/meth.2001.1262 . 11846609

[27] Foldager CB, Munir S, Ulrik-Vinther M, Soballe K, Bunger C, Lind M. Validation of suitable house keeping genes for hypoxia-cultured human chondrocytes. BMC molecular biology. 2009;10:94 10.1186/1471-2199-10-94 ; PubMed Central PMCID: PMCPMC2764705. 19818117 PMC2764705

[28] Dobrian AD, Lieb DC, Cole BK, Taylor-Fishwick DA, Chakrabarti SK, Nadler JL. Functional and pathological roles of the 12- and 15-lipoxygenases. Prog Lipid Res. 2011;50(1):115–31. 10.1016/j.plipres.2010.10.005 ; PubMed Central PMCID: PMCPMC3012140. 20970452 PMC3012140

[29] Moore-Morris T, Guimaraes-Camboa N, Yutzey KE, Puceat M, Evans SM. Cardiac fibroblasts: from development to heart failure. Journal of molecular medicine. 2015;93(8):823–30. 10.1007/s00109-015-1314-y ; PubMed Central PMCID: PMCPMC4512919. 26169532 PMC4512919

[30] Haworth RA, Potter KT, Russell DC. Role of arachidonic acid, lipoxygenase, and mitochondrial depolarization in reperfusion arrhythmias. Am J Physiol Heart Circ Physiol. 2010;299(1):H165–74. 10.1152/ajpheart.00906.2009 . 20435853

[31] Diaz A, Bourassa MG, Guertin MC, Tardif JC. Long-term prognostic value of resting heart rate in patients with suspected or proven coronary artery disease. Eur Heart J. 2005;26(10):967–74. 10.1093/eurheartj/ehi190 . 15774493

[32] Swedberg K, Komajda M, Bohm M, Borer JS, Ford I, Dubost-Brama A, et al Ivabradine and outcomes in chronic heart failure (SHIFT): a randomised placebo-controlled study. Lancet. 2010;376(9744):875–85. 10.1016/S0140-6736(10)61198-1 . 20801500

[33] Honda M, Kiyokawa J, Tabo M, Inoue T. Electrophysiological characterization of cardiomyocytes derived from human induced pluripotent stem cells. Journal of pharmacological sciences. 2011;117(3):149–59. Epub 2011/10/27. . 22027094 10.1254/jphs.11038fp

